# Guidance for Evidence-Informed Policies about Health Systems: Assessing How Much Confidence to Place in the Research Evidence

**DOI:** 10.1371/journal.pmed.1001187

**Published:** 2012-03-20

**Authors:** Simon Lewin, Xavier Bosch-Capblanch, Sandy Oliver, Elie A. Akl, Gunn E. Vist, John N. Lavis, Davina Ghersi, John-Arne Røttingen, Peter Steinmann, Metin Gulmezoglu, Peter Tugwell, Fadi El-Jardali, Andy Haines

**Affiliations:** 1 Norwegian Knowledge Centre for the Health Services, Oslo, Norway, and Health Systems Research Unit, Medical Research Council of South Africa, Cape Town, South Africa; 2 Swiss Tropical and Public Health Institute, Basel, and University of Basel, Basel, Switzerland; 3 EPPI-Centre, Social Science Research Unit, Institute of Education, London, United Kingdom; 4 Department of Medicine, State University of New York at Buffalo, Buffalo, New York, United States of America, and Department of Clinical Epidemiology and Biostatistics, McMaster University, Hamilton, Ontario, Canada; 5 Norwegian Knowledge Centre for the Health Services, Oslo, Norway; 6 McMaster Health Forum, Centre for Health Economics and Policy Analysis, Department of Clinical Epidemiology and Biostatistics, and Department of Political Science, McMaster University, Hamilton, Ontario, Canada; 7 Department of Research Policy and Cooperation, Information, Evidence and Research, World Health Organization, Geneva, Switzerland; 8 Norwegian Knowledge Centre for the Health Services, Oslo, Norway; 9 Swiss Tropical and Public Health Institute, Basel, Switzerland; 10 UNDP/UNFPA/WHO/World Bank Special Programme of Research, Development and Research Training in Human Reproduction, World Health Organization, Switzerland; 11 Department of Medicine, Institute of Population Health, and Department of Epidemiology and Community Medicine, University of Ottawa, Ottawa, Canada; 12 Department of Health Policy and Management, American University of Beirut, Beirut, Lebanon, and McMaster Health Forum, McMaster University, Hamilton, Ontario, Canada; 13 Departments of Social and Environmental Health Research and of Nutrition and Public Health Research, London School of Hygiene and Tropical Medicine, London, United Kingdom

## Abstract

In the third paper in a three-part series on health systems guidance, Simon Lewin and colleagues explore the challenge of assessing how much confidence to place in evidence on health systems interventions.

**Summary Points**

- Assessing how much confidence to place in different types of research evidence is key to informing judgements regarding policy options to address health systems problems.
- Systematic and transparent approaches to such assessments are particularly important given the complexity of many health systems interventions.
- Useful tools are available to assess how much confidence to place in the different types of research evidence needed to support different steps in the policy-making process; those for assessing evidence of effectiveness are most developed.
- Tools need to be developed to assist judgements regarding evidence from systematic reviews on other key factors such as the acceptability of policy options to stakeholders, implementation feasibility, and equity.
- Research is also needed on ways to develop, structure, and present policy options within global health systems guidance.
- This is the third paper in a three-part series in *PLoS Medicine* on health systems guidance.

*This is one paper in a three-part series that sets out how evidence should be translated into guidance to inform policies on health systems and improve the delivery of clinical and public health interventions.*

## Introduction

Health systems interventions establish or modify governance (e.g., licensing of professionals), financial (e.g., health insurance mechanisms) and delivery (e.g., by whom care is provided) arrangements, and implementation strategies (e.g., strategies to change health provider behaviours) within health systems (which consist of “all organisations, people and actions whose primary intent is to promote, restore or maintain health”; see Box S1 for definitions of the terms used in this article). The focus of these interventions is to strengthen health systems in their own right or to get cost-effective programmes and technologies (e.g., drugs and vaccines) to those who need them. Decisions regarding health systems strengthening, including the development of recommendations by policy makers, require evidence on the effectiveness of these interventions, as well as many other forms of evidence. For example, in assessing potential policy options, reviews of economic evaluations and of qualitative studies of stakeholders' views regarding these options might be important (Table S1). Such evidence helps to address questions such as the cost-effectiveness of these options and which options are seen as appropriate by stakeholders.

Assessing how much confidence to place in the types of evidence available on health systems interventions is a key component in informing judgements regarding the use of such interventions for health systems strengthening (Box 1). This paper, which is the third of a three-part series on health systems guidance [1],[2], aims to:

- Illustrate a range of tools available to assess the different types of evidence needed to support different steps in the policy-making process;
- Discuss the GRADE (Grading of Recommendations Assessment, Development and Evaluation) approach to assessing confidence in estimates of effects (“quality of evidence”) and to grading the strength of recommendations on policy options for health systems interventions;
- Discuss factors that are important when developing recommendations on policy options regarding health systems interventions.

**Box 1. Reasons Why It Is Important to Assess How Much Confidence Can Be Placed in Evidence to Guide Decisions on Health Systems Strengthening**

- Users of such evidence almost always draw implicit or explicit conclusions regarding how much confidence to place in evidence
- Such evidence may inform judgements regarding recommendations, including the strength of such recommendations
- Systematic and explicit approaches can be useful in:
  - facilitating critical appraisal of evidence
  - protecting against bias
  - clarifying implementation issues
  - resolving disagreements among stakeholders
  - communicating information regarding the evidence, the judgements made, and recommendations drawn from it
- Systematic and explicit approaches are particularly important given the complexity of many health systems interventions. These interventions may be complex in terms of the number of discrete, active components and the interactions between them; the number of behaviours to which the intervention is directed; the number of organisational levels targeted by the intervention; the degree of flexibility or tailoring permitted in intervention implementation; the level of skill required by those delivering the intervention; and the extent of context dependency [48],[49]. Given the complexity of these interventions, deciding on the contextual relevance of evidence is a crucial component.

Source: adapted from [30].

The first paper in this series makes a case for developing guidance to inform decisions on health systems questions and explores challenges in producing such guidance and how these might be addressed [1]. The second paper explores the links between guidance development and policy development at global and national levels, and examines the range of factors that can influence policy development [2].

In this paper, which like the other two papers is based on discussions of the Task Force on Developing Health Systems Guidance (Box 2; [1],[2]), we focus particularly on the GRADE approach, which provides a transparent and systematic approach to rating the quality of evidence and grading the strength of recommendations [3].

**Box 2. The Task Force on Developing Health Systems Guidance**

- The Task Force on Developing Health Systems Guidance was established in 2009 by the World Health Organization (WHO) to improve its response to requests for guidance on health systems.
- The Task Force consisted of 20 members selected by WHO for their expertise in the field of health systems research and implementation.
- Through a series of face-to-face and virtual meetings, the Task Force provided input to and oversight of the development of a *Handbook for Developing Health Systems Guidance* and to the identification of broader issues that warranted further dialogue and debate [39].
- As part of this process, the Task Force and the Handbook developers reviewed approaches to developing clinical guidelines and the instruments used for clinical guideline development.
- The Task Force suggested ways in which some of these approaches and instruments could be adapted for use in the development of health systems guidance and indicated where there were important differences between these approaches.
- The writing group for this paper further considered the issues raised in these discussions and produced a first draft of the manuscript for comment by the Task Force.
- This paper, and the other two in the series [1],[2], were finalised after several iterations of comments by the Task Force and external reviewers.

## Tools to Assess the Evidence Needed to Support the Policy-Making Process for Health Systems Strengthening

Well-conducted systematic reviews [4] can be used to identify the best available evidence to inform judgements about the effects of policy options and to inform other key steps within the policy-making process (Table S1). As discussed in the second paper of this series [2], users need to be able to assess the quality of evidence presented in such reviews in relation to each step of the policy-making process. For example, when defining the problem and the need for intervention, tools are required to assess the confidence we can place in evidence from reviews of studies highlighting different ways of conceptualising the problem (e.g., reviews of studies of people's experiences of the problem) [5]. When assessing potential policy options, tools are needed to assess the confidence that can be placed in, for example, studies assessing impact (e.g., reviews of effectiveness studies). Similarly, when identifying implementation considerations, tools are required to assess the confidence that can be placed in reviews of factors affecting implementation.

Many tools are available to assess the risk of bias in individual studies of the effects of interventions [6] and to appraise individual qualitative studies [7]. Tools are also available to assess the quality of evidence synthesised in systematic reviews [6]. Such tools need to be appropriate to the types of studies included in the review and generic enough to be applicable across a range of questions, and must allow meaningful conclusions to be drawn regarding the quality of the included evidence. Judgements on how much confidence can be placed in the evidence from a review need to be distinguished from judgements about how well the review was conducted (i.e., its reliability). Tools have been developed to assess the reporting of systematic reviews and meta-analyses (e.g., the PRISMA checklist [8]) and to assess their methodological quality or reliability (e.g., the SUPPORT tools [9] and AMSTAR [10]) (Table 1). However, we focus here on tools to assess how much confidence can be placed in the evidence identified and presented in those reviews.

**Table 1:** Commonly used tools to assess systematic reviews and their findings and to assess clinical guidelines.

*Systematic Reviews*	
SUPPORT (SUPporting Policy Relevant Reviews and Trials) tool [9]	A tool to assess how much confidence to place in the methodological quality of a systematic review, and designed for reviews of health systems interventions
AMSTAR (A measurement tool for the “assessment of multiple systematic reviews”) [10]	A tool designed to assess the methodological quality of a systematic review. This tool has not been designed specifically to assess how much confidence to place in reviews of health systems interventions
GRADE (Grading of Recommendations Assessment, Development and Evaluation) [41]	An approach to assess the quality of evidence
PRISMA (Preferred Reporting Items for Systematic Reviews and Meta Analyses) [8]	A tool to assess the reporting of systematic reviews and meta-analyses
SUPPORT applicability tool [33] and Wang applicability and transferability tool [34]	Tools to assist in assessing the applicability of the findings of a systematic review to a specific setting

*Guidelines*	
AGREE (Appraisal of Guidelines for Research and Evaluation) [42]	A tool to evaluate the process of practice guideline development and the quality of reporting
GRADE [41]	An approach to grade the strength of recommendations

### Assessing How Much Confidence to Place in the Findings of Reviews of the Effects of Policy Options

Tools to assess how much confidence to place in review findings are most developed for systematic reviews of evidence on effectiveness. The GRADE approach is one such tool, but many others are available [11]. Within GRADE, the quality of evidence derived from a systematic review is related to the quality of the included studies and to a range of other factors (Table S2). This approach has many strengths (Table S3) and is now used increasingly by international organizations, including the World Health Organization (WHO), the Cochrane Collaboration, and several agencies developing guidelines [3]. (Also see http://www.gradeworkinggroup.org.) We will discuss the application of the GRADE approach to assess the quality of evidence and the strength of recommendations for health systems interventions later.

### Assessing How Much Confidence to Place in the Findings of Reviews of Questions Other Than Effects

Tools to assist judgements on how much confidence to place in the findings of reviews of questions other than effects that are relevant to the policy-making process are at an early stage of development. Such questions include stakeholders' values and preferences and the feasibility of interventions (Table S1). For some of these issues, judgements might be informed by systematic reviews of qualitative studies, together with local evidence [12]. Where reviews seek a qualitative answer to understand the nature of a problem, quality appraisal aims to assess the coherence of the resulting explanation, possibly across different contexts. Although quality criteria for individual qualitative research studies commonly consider the methods of each study and the credibility and richness of their findings [7],[13],[14], thus far tools for assessing the quality of systematic reviews of qualitative research have not considered the credibility and richness of findings. A potential tool for doing this is proposed in Table S4.

Reviews exploring factors affecting the implementation of options might employ mixed methods syntheses (i.e., syntheses of both qualitative and quantitative evidence) such as realist synthesis [15], which explores the explanatory theories implicit in existing programmes or policies, or framework synthesis, which provides a highly structured, deductive approach to data analysis drawing on an existing model or framework (for example, [16]–[18]). Quality appraisal then focuses on the confidence that can be placed in each conclusion drawn from individual studies [19]. Grading the evidence as a whole can take into account the number and context of the studies contributing to each conclusion, and the appropriateness of their methods for drawing that conclusion (for illustration, see [16]). A single study might refute or qualify a theory, but multiple studies together contribute to strengthening a theory. As yet, there are no tools for appraising how well mixed methods reviews have synthesised studies to draw conclusions about the advantages or disadvantages of policy options.

Resource use is another key issue, and tools are available to assess the reliability of reviews of economic studies [20]. In addition, GRADE provides guidance on how to incorporate considerations of resource use into recommendations [21].

## The GRADE Approach to Assessing Confidence in the Estimates of Effects for Health Systems Interventions

The GRADE approach clearly separates two issues: the quality of the evidence and the strength of recommendations. Quality of evidence is only one of several factors considered when assessing the strength of recommendations.

Within the context of a systematic review, GRADE defines the quality of evidence as the extent to which one can be confident in the estimate of effect. Within the context of guidelines or guidance, GRADE defines the quality of evidence as the confidence that the effect estimate supports a particular recommendation. The degree of confidence is a continuum but, for practical purposes, it is categorised into high, moderate, low, and very low quality (Table S5).

Evidence on the effectiveness of health systems interventions raises a number of challenges that may, in turn, influence assessments of the quality of this evidence and the development of recommendations using the GRADE approach. Firstly, while experimental studies (including pragmatic randomised trials [22]) are feasible for some health systems interventions, for others (particularly those related to governance and financial arrangements), evidence may come mainly from observational studies, including evaluations of national or state-wide programmes [23],[24]. Secondly, evaluations of health systems interventions often use clustered designs and these are frequently poorly conducted, analysed, and reported [25],[26]. Thirdly, health systems interventions tend to measure proxy outcomes, such as the use of services or the uptake of an incentive. Evidence users need to decide whether there is sufficiently strong evidence of a relationship between the proxy outcome and the desired health outcome. The development of an outcomes framework to assist in assessing interventions (for an example, see [27],[28]) may help those developing guidance decide whether proxy outcomes are sufficient. Finally, poorly described health systems and political systems factors and implementation considerations may make it difficult to develop contextualised recommendations on policy options [29]. GRADE attempts to make the judgements regarding these issues systematic and transparent.

## Developing Recommendations on Policy Options for Consideration Regarding Health Systems Interventions

Moving from evidence to recommendations on options for consideration often necessitates the interpretation of factors other than evidence. In most cases, these interpretations require judgments, making it important to be transparent, particularly given that recommendations will sometimes need to consider multiple complex health systems interventions, each with its own assessment of quality of evidence. Another challenge is the additional complexity of assessing the wide range of health system and political system factors that will influence the choice and implementation of options for addressing a health system problem in different settings (see the other papers in this series [1],[2]). For example, a health systems problem may involve a wide range of stakeholders, each with views regarding the available options. In addition, health systems interventions may have system-wide effects that vary across settings. Consequently, rather than making a single recommendation as in clinical guidelines, it may be more useful for health systems guidance to set out the evidence and outline a range of options, appropriate to different settings, to address a given health systems problem. These options may, in turn, feed into deliberative or decision-making processes at national or sub-national levels, as discussed later and elsewhere in this series [2].

Tools such as GRADE assist in grading the strength of a recommendation regarding options [30],[31] and can be applied to health systems interventions, but may benefit from explicitly including some additional factors (Box 3). Further research is needed to explore the usefulness of these additional factors but, in general, any tool used to guide the development of recommendations should aim to improve transparency by explicitly describing the factors, and their interpretation, that contributed to the development of recommendations.

**Box 3. Factors That May Inform Decisions about the Strength of Recommendations Regarding Policy Options**

*GRADE factors (adapted from* [*30*] *):*

- Whether there is uncertainty about the balance of benefits versus harms and burdens
- The quality of the evidence from the systematic review (very low, low, moderate, high)
- Whether there is uncertainty or variability in values and preferences among stakeholders
- Whether there is uncertainty about whether the net benefits are worth the costs or about resource use
- Whether there is uncertainty about the feasibility of the intervention (or about local factors that influence the translation of evidence into practice, including equity issues)

*Additional factors that it may be useful to consider for health systems interventions:*

- Ease of implementation at the systems level, including governance arrangements (e.g., changes needed in regulations), financial arrangements (e.g., the extent to which the options fit with financing models within settings), and implementation strategies (e.g., how to provide the skills and experience needed among implementers or facilitators)
- Socio-political considerations, e.g., how the proposed options relate to existing policies, values within the political system in relation to issues such as equity or privatisation, and economic changes

Within the GRADE approach, recommendations reflect the degree of confidence that the desirable effects of applying a recommendation outweigh the undesirable effects. Specifically, a strong recommendation implies confidence that the desirable effects of applying a recommendation outweigh the undesirable effects, whereas a conditional/qualified/weak recommendation suggests that the desirable effects of applying a recommendation probably outweigh the undesirable effects, but there is uncertainty.

GRADE attempts to make all judgments regarding the factors that are considered in developing recommendations transparent (by documenting these judgments) and systematic (by using the same approach across all the questions being considered by the guideline). Tables 2, S6, and S7 provide illustrations of the application of the GRADE approach to health systems interventions involving delivery and financial arrangements and implementation strategies, respectively, and show how guidance on health systems interventions might outline a range of options appropriate to different settings—an approach on which further research is needed. In common with other grading systems, GRADE does not yet provide guidance on how to assess the level of confidence that can be placed in evidence on “acceptability” or “feasibility”. Conventionally, these judgements have been made by consensus among the guideline panel, which needs to include individuals with expertise and experience relevant to the guideline questions. Further work is needed to develop a formal way of assessing the quality of such evidence.

**Table 2:** Example of factors affecting decisions about strength of recommendations—Lay or community health workers to reduce childhood mortality.

Population: Children in high mortality settingsIntervention: Lay health workers (LHWs) delivering health promotion, treatment, and referral interventionsComparison: No LHW intervention / usual careOutcome: Childhood mortality		
Key factors—is there uncertainty regarding:	Decision regarding whether there is uncertainty (yes / no)	Explanation of the decision made
Quality of evidence	Yes	The use of LHWs in maternal and child health programmes may lead to fewer deaths among children under five (low quality evidence—GRADE). In addition, the use of LHWs probably leads to an increase in the number of women who breastfeed and to the number of children who have their immunisation schedule up to date (moderate quality evidence for both outcomes— GRADE). These additional outcomes are also related to mortality reduction
Balance of benefits versus harms and burdens	Yes	Potentially important benefits (mortality reduction) but confidence interval also includes harm. Additional evidence on LHWs suggests effectiveness, e.g., LHWs associated with increased uptake of interventions of proven cost-effectiveness (immunisation, breastfeeding)
Acceptability	Yes	• Some evidence that LHWs acceptable to service users and used widely• Varied acceptability to other services providers in different settings (e.g., [43],[44])
Resource use	Yes	Potentially large investment needed over long period but alternatives likely to be more costly
Feasibility (or local factors that influence the translation of evidence into practice)	Yes	There may be constraints to scaling up trained LHWs and supporting them, but it is even less feasible to scale up professional cadres. There are a number of well-documented examples of LHW programmes that have been taken to scale for which monitoring has suggested some positive outcomes, e.g., in Ethiopia and Pakistan [45],[46]
**Recommended options for consideration** *This assessment of evidence within a wider health system context might result in the following recommended options for consideration:*• Option 1: Where child mortality is high; an infrastructure for LHWs can be developed rapidly; it is unlikely that the numbers of other cadres could be expanded; and this cadre is acceptable to other providers and to service users and has strong political support:○ Strong recommendation to implement LHWs to reduce childhood mortality (i.e., there is confidence that the desirable effects of LHWs delivering interventions to reduce childhood mortality outweigh the undesirable effects).• Option 2: Where child mortality is high; LHWs are acceptable to other providers and to service users; but governance and financing mechanisms for LHWs will need to be established, and there is little experience of running such programmes and uncertainty among policy makers:○ Conditional recommendation to implement LHWs to reduce childhood mortality (i.e., the desirable effects of LHWs delivering interventions to reduce childhood mortality probably outweigh the undesirable effects, but there is uncertainty).• Option 3: Where child mortality is moderate; an infrastructure for LHWs can be developed rapidly; but there is evidence that the scaling up of LHWs may be challenged by health care professionals:○ Conditional recommendation to implement LHWs to reduce childhood mortality, dependent on the ability to overcome professional opposition (i.e., the desirable effects of LHWs delivering interventions to reduce childhood mortality probably outweigh the undesirable effects, but there is uncertainty).		

Source: This table draws on evidence from [47].

### Challenges in Moving from an Assessment of the Quality of Evidence to Making Recommendations on Policy Options

The move from an assessment of the quality of evidence to making recommendations on policy options involves a number of challenges. Firstly, assessments of the strength of a recommendation may require a detailed understanding of the evidence creation and evaluation process that is not always available. Secondly, categorising recommendations as “strong” and “weak” can raise difficulties. Panels developing global guidance may, for example, be reluctant to make “weak” recommendations in case policy makers fail to respond to such recommendations because they assume they are equivalent to “no recommendation”. Thirdly, the quality of evidence is typically assessed for two alternative policy options in tools such as GRADE, while many health system (and, indeed, many clinical decisions) involve multiple interventions, which adds to the complexity of interpretation and decision-making and makes it even more important to be transparent.

How might some of these challenges be addressed? Methodological expertise is needed to conduct and interpret systematic reviews and perform assessments using tools such as GRADE. Health systems guidance panels therefore need to be supported by methodology experts. Moreover, the outputs of these tools need to be “translated” into appropriate language and formats to ensure that they can be interpreted and used correctly by the panel. Research is under way within initiatives such as the DECIDE (Developing and Evaluating Communication strategies to support Informed Decisions and practice based on Evidence; http://www.decide-collaboration.eu) collaboration on ways of presenting information on GRADE assessments and policy options to policy makers.

### Additional Challenges

There are other wider challenges involved in making recommendations on policy options regarding health systems interventions. Firstly, the tools used to assess the quality of evidence and develop recommendations need to be able to accommodate the wide range of study designs that is used to assess the effectiveness of health system interventions. This is possible within GRADE. Secondly, tools need to be developed to inform judgements on how much confidence to place in the other forms of evidence (e.g., evidence on acceptability) that are needed to develop recommendations regarding health systems interventions [32].

Finally, international standard-setting organisations, such as WHO, have to formulate recommendations that are applicable at a global level. However, as noted earlier, creating global recommendations on health systems questions can be difficult because of important variations in context-specific factors that influence the applicability of interventions at national and sub-national levels [2],[33]. An approach that should help to link guidance development at the global level with policy development at the national level is outlined in the second paper of this series [2].

Where it is useful to make recommendations at the global level, those developing guidance may choose to outline policy options rather than a single recommendation. Such options may encompass one or more questions and may be based on the range of interventions considered in relation to these questions. Health and political systems factors could be taken into account by linking specific options to these factors. For example, the options may describe variations in the intervention content and method of implementation, based on the evaluations that have been conducted in different settings. Further work is needed to explore how policy makers might interpret and select policy options outlined in global guidance. However, one useful approach might be to provide national decision makers with tools to assist them in making recommendations appropriate to their setting. Several such tools are available [33],[34] or in development (see http://www.decide-collaboration.eu/work-packages-strategies). Importantly, global guidance should always indicate the factors that should be considered to assess the implications of variations in intervention, context, and other conditions. Decision models may be useful in exploring the effects of these variations (for example, [35],[36]). Given the often low quality evidence available regarding policy options for health systems problems, it is also likely that in many cases the recommended option(s) will need to be evaluated.

### Presenting Evidence Regarding Contextual and Implementation Issues to Guidance Panels and Policy Makers

The best way to communicate evidence on contextual and implementation issues related to health systems and political systems to guidance panels and policy makers to inform their judgements about the strength of recommended options is currently unclear. Related work on summary of findings tables for systematic reviews of effects and evidence summaries for policy makers has illustrated the importance of paying attention to both format and content in developing useful and understandable presentation approaches [37],[38]. The *Handbook for Developing Health Systems Guidance* sets out an approach for presenting this type of evidence to stakeholders in a user-friendly evidence profile [39]. Similarly, the second paper in this series describes the wider features of health and political systems that may need to be assessed to inform decision-making [2].

If we want to ensure that guidance panels and policy makers use evidence to inform judgements about the strength of recommended options, more research is needed to develop and test approaches (including visual formats) for presenting the available evidence to such groups. In addition, efforts are needed to build the capacity of policy makers to use evidence to inform their decisions [2],[12],[40].

## Conclusions

Useful tools are available for grading quality of evidence and strength of recommendations on policy options regarding health systems interventions, but several challenges need to be addressed. Firstly, these tools involve judgements, and these need to be made systematically and transparently. Secondly, for many health systems questions, evidence is still likely to be of low quality. Better quality research in these areas is needed and would allow guidance panels to have more confidence in the evidence and to issue stronger recommendations. Thirdly, research is needed on ways to develop, structure, and present policy options for consideration within global health systems guidance. These options need to include evidence on health and political system factors and implementation considerations, and tools to assess such evidence need to be refined. Finally, greater attention needs to be given to how guidance on health systems interventions may be implemented at the local level.

## Supporting Information

Alternative Language Summary Points S1 **Translation of the Summary Points into Spanish by Xavier Bosch-Capblanch**
(DOC)

Alternative Language Summary Points S2 **Translation of the Summary Points into French by Bruno Clary, William Lenoir, and Lise Beck**
(DOC)

Alternative Language Summary Points S3 **Translation of the Summary Points into Portuguese by Bruno Viana**
(DOC)

Alternative Language Summary Points S4 **Translation of the Summary Points into Arabic by Fadi El-Jardali**
(DOC)

Box S1 **Definitions of terms used in this paper**
(PDF)

Table S1 **Types of systematic reviews needed for different steps in the policy-making process and the availability of tools to assess how much confidence can be placed in the evidence presented in these reviews**
(PDF)

Table S2 **GRADE criteria for assessing the quality of evidence for each important outcome assessed in a systematic review of effects**
(PDF)

Table S3 **Strengths of the GRADE approach**
(PDF)

Table S4 **Assessing how much confidence can be placed in the findings of systematic reviews of qualitative studies**
(PDF)

Table S5 **Definitions of the quality of evidence categories within GRADE**
(PDF)

Table S6 **Example of factors affecting decisions about strength of recommendations—Changes in user fees in low- and middle-income countries**
(PDF)

Table S7 **Example of factors affecting decisions about strength of recommendations—Continuing education programmes for rural health workers to support their retention**
(PDF)

## Abbreviations

GRADE: Grading of Recommendations Assessment, Development and Evaluation
WHO: World Health Organization
