# Supplementary material for: Guidance for Evidence-Informed Policies about Health Systems: Assessing How Much Confidence to Place in the Research Evidence
Source: PLoS Med. 2012 Mar 20;9(3):e1001187. doi: 10.1371/journal.pmed.1001187 (PMC3308931; doi:10.1371/journal.pmed.1001187)
Supplement: Alternative Language Summary Points S4 — Translation of the Summary Points into Arabic by Fadi El-Jardali (DOC) [file pmed.1001187.s004.doc]

Guidance for Evidence-Informed Policies about Health Systems: Assessing How Much Confidence to Place in the Research Evidence

**نقاط موجزة**

- إن تقييم حجم الثقة الذي يجب إعطاؤه للأنواع المختلفة من البيّنات والأدلة البحثية ضرورة مفتاحية في جعل الأحكام بخصوص خيارات السياسات أكثر وعياً واطّلاعاً في مجال معالجة مشاكل الأنظمة الصحية.

- إن المقاربات المنهجية والشفافة لمثل هذه التقييمات هي بالأخص ذات الأهمية، نظراً لدرجة التعقيد التي تحيط بالعديد من تدخّلات وإجراءات الأنظمة الصحّية.

- هناك أدوات مفيدة متوفرة لتقييم حجم الثقة الواجب إعطاؤها للأنواع المختلفة من البيّنات والأدلة البحثية المطلوبة لدعم الخطوات المختلفة من عملية صناعة القرار؛ ولعل هذه المتخصصة بتقييم درجة الفعالية هي الأكثر تطوراً وتوفراً في هذا المجال.

- هناك حاجة لوضع وتطوير أدوات مساعدة في مجال إعطاء الأحكام بالنظر إلى الأدلة والبيّنات من المراجعات المنهجية بخصوص عوامل رئيسية أخرى مثل درجة قبول خيارات السياسات لدى أصحاب الشأن المعنيين، وإمكانية التطبيق ودرجة صعوبته/سهولته، والمساواة.

- هناك أيضاً حاجة إلى مزيد من الأبحاث حول طرق وضع، وتطوير، وبنية، وتقديم، خيارات السياسات في إطار تقديم المشورة والإرشادات للأنظمة الصحية على المستوى الدولي/ العالمي.
