# Supplementary material for: Guidance for Evidence-Informed Policies about Health Systems: Assessing How Much Confidence to Place in the Research Evidence
Source: PLoS Med. 2012 Mar 20;9(3):e1001187. doi: 10.1371/journal.pmed.1001187 (PMC3308931; doi:10.1371/journal.pmed.1001187)
Supplement: Box S1 — Definitions of terms used in this paper (PDF) [file pmed.1001187.s005.pdf]

Box S1: Definitions of terms used in this paper

- Health systems: consists of all organizations, people and actions whose primary intent is to promote, restore or maintain health [1]
- Health systems interventions: establishment or modifications to governance, financial and delivery arrangements within health systems, as well as implementation strategies within health systems, the focus of which is to strengthen health systems in their own right or to get cost-effective programs, services and technologies (e.g., drugs, vaccines and diagnostic tests) to those who need them (and hence where the benefits and harms are not just measured at the level of individuals)
    - Such interventions can be distinguished from clinical interventions (e.g., antenatal visits and drugs) and from public health interventions (immunizations and health promotion campaigns)
- Health systems guidance: systematically developed statements created at the global or national level to assist decisions about appropriate options for addressing a health system problem in a range of settings as well as to assist with implementation and with monitoring and evaluation
    - Health systems guidance could take many forms, but one example could be statements such as: 'it would be reasonable to pursue option A in order to address health system problem B in circumstances such as those faced in countries C and D, taking into account health system features E and F and political system features G and H, whereas it would be less reasonable to pursue option X in such countries given....'
    - In the absence of research evidence, health systems guidance could identify the nature of any monitoring and evaluation that would be needed
    - A health systems analysis and a political analysis would be needed in addition to the assessment of the health system problem under consideration
- Policy brief (evidence brief [2]): systematically developed statements created at the national or sub-national level to assist decisions about appropriate options for addressing a health system problem in that specific setting, and that may also assist with implementation and with monitoring and evaluation, and that may be used in national guidance development processes or in policy development processes
- Policy dialogue (stakeholder dialogue [3]): systematically planned processes organized at the national or sub-national level to solicit the views, experiences and tacit knowledge of those who will be involved in or affected by decisions about appropriate options for addressing a health system problem in that specific setting, and a summary of which may be used in policy development processes
- Evidence-Informed Policy Network (EVIPNet): A partnership among policymakers, stakeholders (including civil society) and researchers that promotes the systematic use of research evidence in policymaking about health systems. While focused typically at the national level and occasionally at the sub-national, EVIPNets are embedded within a regional and global collaborative social network supported by WHO [4]

Source: This box is taken from the second paper in this series [5].

References

1. WHO (2008) Everybody's Business: Strengthening Health Systems to Improve Health Outcomes: WHO's Framework for Action. Geneva, Switzerland: World Health Organization.

2. Lavis JN, Permanand G, Oxman AD, Lewin S, Fretheim A (2009) SUPPORT Tools for evidence-informed health Policymaking (STP) 13: Preparing and using policy briefs to support evidence-informed policymaking. Health Res Policy Syst 7 Suppl 1: S13.

3. Lavis JN, Boyko JA, Oxman AD, Lewin S, Fretheim A (2009) SUPPORT Tools for evidence-informed health Policymaking (STP) 14: Organising and using policy dialogues to support evidence-informed policymaking. Health Res Policy Syst 7 Suppl 1: S14.

4. Hamid M, Bustamante-Manaog T, Truong VD, Akkhavong K, Fu H, Ma Y, Zhong X, Salmela R, Panisset U, Pang T (2005) EVIPNet: translating the spirit of Mexico. Lancet 366: 1758-1760.

5. Lavis JN, Røttingen JA, Bosch-Capblanch X, Atun R, El-Jardali F, Gilson L, Lewin S, Oliver S, Ongolo-Zogo P, Haines A (2012) Guidance for evidence-informed policies about health systems: linking guidance development to policy development. PLoS Med 9: e1001187. doi:10.1371/journal.pmed.1001187
