# Supplementary material for: Guidance for Evidence-Informed Policies about Health Systems: Assessing How Much Confidence to Place in the Research Evidence
Source: PLoS Med. 2012 Mar 20;9(3):e1001187. doi: 10.1371/journal.pmed.1001187 (PMC3308931; doi:10.1371/journal.pmed.1001187)
Supplement: Table S1 — Types of systematic reviews needed for different steps in the policy-making process and the availability of tools to assess how much confidence can be placed in the evidence presented in these reviews (PDF) [file pmed.1001187.s006.pdf]

Table S1: Types of systematic reviews needed for different steps in the policymaking process and the availability of tools to assess how much confidence can be placed in the evidence presented in these reviews [a,b]

Steps in the policymaking process	Policy question	Sources of appropriate evidence to address the questions	Tools for assessing the amount of confidence that can be placed in evidence from *systematic reviews* of different kinds of evidence[c,d,e]
Defining and framing the problem[f]	What is the need for intervention, in terms of the nature, magnitude and framing of the problem?	• Reviews of observational studies addressing the nature and magnitude of the problem (e.g. surveys, studies based on routine data to assess prevalence or burden of disease and studies of equity across the most disadvantaged– least disadvantaged groups)<br>• Reviews of qualitative studies of views and experiences regarding the problem	• No widely accepted tool but the general principles underlying tools such as GRADE may be useful (see [1] and Table S3)<br>• No widely accepted tool (see Table S4)
Assessing potential policy options	What is the appropriate set of policy options to address the problem and what are the effects of these options?	• Reviews of studies of the effectiveness of health systems interventions - experimental, non-experimental<br>• Reviews of economic evaluations of health systems interventions<br>• Reviews of qualitative studies of views and experiences regarding health systems interventions	• GRADE, US Preventive Services Task Force, NICE [2–4] (for experimental & non-experimental studies)<br>• Quality tool described in [5]; GRADE [6]<br>• No widely accepted tool (see Table S4)
Identifying implementation considerations for selected policy options	What are the potential factors affecting the successful implementation of the policy options?	• Reviews of effectiveness studies of implementation strategies and of equity across the most disadvantaged– least disadvantaged groups)<br>• Reviews of qualitative studies of the acceptability of health systems interventions<br>• Reviews of process evaluations of implementation strategies, which may use mixed methods	• GRADE<br>• No widely accepted tool (see Table S4)<br>• No widely accepted tool

Source: Adapted from [7,8]

[a] This table is not based on a systematic review of available tools, but rather on discussions within the Task Force. It is therefore likely that some available tools have been missed.

[b] This table does not intend to suggest that policy making typically follows these steps in a linear fashion but rather uses these steps to illustrate the different types of systematic reviews that may be needed during the policy process. Within policy making processes, these steps may occur simultaneously and iteratively and evidence needs to be made available as opportunities arise to use this evidence.

[c] In applying these tools, the review question also needs to be taken into account. For instance, have appropriate review methods been used to address the question?

[d] A number of other tools are available to improve the reporting of systematic reviews and meta-analyses of a range of studies or to assess the quality of reporting (e.g. [9–12]). These are not listed here as they are not intended to assess how much confidence can be placed in the findings of a review.

[e] A number of other tools to appraise the quality of evidence from systematic reviews are used by specific guideline development agencies and institutions, e.g. EPPI-Centre (http://eppi.ioe.ac.uk/cms/); JBI (http://www.joannabriggs.edu.au/about/home.php). Other tools which are (or have been) regularly employed are introduced and compared in Atkins 2004 [13].
[f] Systematic reviews may not be needed for all questions related to defining the problem – see the second paper in this series for further discussion of this [14].

References

1. Guyatt G, Oxman AD, Akl EA, Kunz R, Vist G, Brozek J, Norris S, Falck-Ytter Y, Glasziou P, DeBeer H, Jaeschke R, Rind D, Meerpohl J, Dahm P, Schunemann HJ (2011) GRADE guidelines: 1. Introduction-GRADE evidence profiles and summary of findings tables. J Clin Epidemiol 64: 383-394.

2. Guyatt G, Oxman AD, Akl EA, Kunz R, Vist G, Brozek J, Norris S, Falck-Ytter Y, Glasziou P, DeBeer H, Jaeschke R, Rind D, Meerpohl J, Dahm P, Schunemann HJ (2011) GRADE guidelines: 1. Introduction-GRADE evidence profiles and summary of findings tables. J Clin Epidemiol 64: 383-394.

3. Sawaya GF, Guirguis-Blake J, LeFevre M, Harris R, Petitti D (2007) Update on the methods of the U.S. Preventive Services Task Force: estimating certainty and magnitude of net benefit. Ann Intern Med 147: 871-875.

4. NICE (2011) Methods for the development of NICE public health guidance (second edition). London, United Kingdom: National Institute for Health and Clinical Excellence.

5. Jefferson T, Demicheli V, Vale L (2002) Quality of systematic reviews of economic evaluations in health care. JAMA 287: 2809-2812.

6. Guyatt GH, Oxman AD, Kunz R, Jaeschke R, Helfand M, Liberati A, Vist GE, Schunemann HJ (2008) Incorporating considerations of resources use into grading recommendations. BMJ 336: 1170-1173.

7. Lavis JN (2009) How can we support the use of systematic reviews in policymaking? PLoS Med 6: e1000141.

8. Lavis JN, Oxman AD, Lewin S, Fretheim A (2009) SUPPORT Tools for evidence-informed health Policymaking (STP). Health Res Policy Syst 7 Suppl 1: I1.

9. Stroup DF, Berlin JA, Morton SC, Olkin I, Williamson GD, Rennie D, Moher D, Becker BJ, Sipe TA, Thacker SB (2000) Meta-analysis of observational studies in epidemiology: a proposal for reporting. Meta-analysis Of Observational Studies in Epidemiology (MOOSE) group. JAMA 283: 2008-2012.

10. von Elm E, Altman DG, Egger M, Pocock SJ, Gotzsche PC, Vandenbroucke JP (2007) The Strengthening the Reporting of Observational Studies in Epidemiology (STROBE) statement: guidelines for reporting observational studies. PLoS Med 4: e296.

11. Wong WC, Cheung CS, Hart GJ (2008) Development of a quality assessment tool for systematic reviews of observational studies (QATSO) of HIV prevalence in men having sex with men and associated risk behaviours. Emerg Themes Epidemiol 5: 23.

12. Des Jarlais DC, Lyles C, Crepaz N (2004) Improving the reporting quality of nonrandomized evaluations of behavioral and public health interventions: the TREND statement. Am J Public Health 94: 361-366.

13. Atkins D, Eccles M, Flottorp S, Guyatt GH, Henry D, Hill S, Liberati A, O'Connell D, Oxman AD, Phillips B, Schunemann H, Edejer TT, Vist GE, Williams JW, Jr. (2004) Systems for grading the quality of evidence and the strength of recommendations I: critical appraisal of existing approaches The GRADE Working Group. BMC Health Serv Res 4: 38.

14. Lavis JN, Røttingen JA, Bosch-Capblanch X, Atun R, El-Jardali F, Gilson L, Lewin S, Oliver S, Ongolo-Zogo P, Haines A (2012) Guidance for evidence-informed policies about health systems: linking guidance development to policy development. PLoS Med 9: e1001187. doi:10.1371/journal.pmed.1001187
