# Supplementary material for: Guidance for Evidence-Informed Policies about Health Systems: Assessing How Much Confidence to Place in the Research Evidence
Source: PLoS Med. 2012 Mar 20;9(3):e1001187. doi: 10.1371/journal.pmed.1001187 (PMC3308931; doi:10.1371/journal.pmed.1001187)
Supplement: Table S2 — GRADE criteria for assessing the quality of evidence for each important outcome assessed in a systematic review of effects (PDF) [file pmed.1001187.s007.pdf]

Table S2: GRADE criteria for assessing the quality of evidence for each important outcome assessed in a systematic review of effects [a]

**Study design**	**Initial quality of a body of evidence**	**Lower if**	**Higher if [1]**	**Quality of a body of evidence**
**Randomised trials[b]**	**High**	**Risk of bias**<br>**Inconsistency**<br>**Indirectness**<br>**Imprecision**<br>**Publication bias**	**Large effect**<br>**Dose response**<br>**All plausible confounding & bias**<br>- Would reduce a demonstrated effect<br>- Would suggest a spurious effect if no effect was observed	**High**<br>⊕⊕⊕⊕
				**Moderate**<br>⊕⊕⊕○
**Observational studies[c]**	**Low**			**Low**<br>⊕⊕○○
				**Very Low**<br>⊕○○○

Source: [2]

[a] Guidance on the use of the GRADE approach is available elsewhere [3,4].

[b] Randomised trials enter the GRADE system as high quality evidence as this design is assumed to be at lower risk of bias than observational studies. The quality may be raised or lowered in relation to the categories outlined in columns 3 and 4.

[c] Observational studies enter the GRADE system as low quality evidence as these designs are assumed to be at higher risk of bias than randomised trials. The quality may be raised or lowered in relation to the categories outlined in columns 3 and 4.

References

1. Guyatt GH, Oxman AD, Sultan S, Glasziou P, Akl EA, Alonso-Coello P, Atkins D, Kunz R, Brozek J, Montori V, Jaeschke R, Rind D, Dahm P, Meerpohl J, Vist G, Berliner E, Norris S, Falck-Ytter Y, Murad MH, Schunemann HJ (2011) GRADE guidelines: 9. Rating up the quality of evidence. J Clin Epidemiol 64: 1311-1316.

2. Guyatt G, Oxman AD, Akl EA, Kunz R, Vist G, Brozek J, Norris S, Falck-Ytter Y, Glasziou P, DeBeer H, Jaeschke R, Rind D, Meerpohl J, Dahm P, Schunemann HJ (2011) GRADE guidelines: 1. Introduction-GRADE evidence profiles and summary of findings tables. J Clin Epidemiol 64: 383-394.

3. Guyatt GH, Oxman AD, Vist GE, Kunz R, Falck-Ytter Y, Alonso-Coello P, Schunemann HJ (2008) GRADE: an emerging consensus on rating quality of evidence and strength of recommendations. BMJ 336: 924-926.

4. Guyatt GH, Oxman AD, Schunemann HJ, Tugwell P, Knottnerus A (2011) GRADE guidelines: a new series of articles in the Journal of Clinical Epidemiology. J Clin Epidemiol 64: 380-382.
