# Supplementary material for: Guidance for Evidence-Informed Policies about Health Systems: Assessing How Much Confidence to Place in the Research Evidence
Source: PLoS Med. 2012 Mar 20;9(3):e1001187. doi: 10.1371/journal.pmed.1001187 (PMC3308931; doi:10.1371/journal.pmed.1001187)
Supplement: Table S3 — Strengths of the GRADE approach (PDF) [file pmed.1001187.s008.pdf]

Table S3: Strengths of the GRADE approach

Issue	Strengths of GRADE
**Definitions**	Provides explicit definitions and grades of quality of evidence and strength of recommendations. The framework also makes clear what the implications of different grades are.
**Process**	Requires systematic, explicit, and transparent judgments. This approach should reduce risks of bias that can arise when judgments are made implicitly or non-systematically.
**Factors affecting quality of evidence**	Systematically considers factors that may affect assessment of the quality of evidence (see Table S2).
**Factors affecting strength of recommendation**	Systematically considers factors that may affect assessment of the strength of recommendations (see Box 3).
**Focus on the important outcomes**	Systematically assesses the quality of evidence for each of the important outcomes, including important outcomes with no or limited evidence.
**Summary tables**	GRADE evidence profiles and summary of findings tables report in a consistent and standardized way both quality assessment and summary of findings. This ensures that all panel members base their judgments on the same information. It also makes this information available to guideline users.
**Wide adaptation**	GRADE members come from a wide range of organizations working on development and evaluation. The system builds on previous experience. It has been widely adopted.

Source: This table draws on [1]

References

1. Guyatt GH, Oxman AD, Vist GE, Kunz R, Falck-Ytter Y, Alonso-Coello P, Schunemann HJ (2008) GRADE: an emerging consensus on rating quality of evidence and strength of recommendations. BMJ 336: 924-926.
