# Supplementary material for: Guidance for Evidence-Informed Policies about Health Systems: Assessing How Much Confidence to Place in the Research Evidence
Source: PLoS Med. 2012 Mar 20;9(3):e1001187. doi: 10.1371/journal.pmed.1001187 (PMC3308931; doi:10.1371/journal.pmed.1001187)
Supplement: Table S4 — Assessing how much confidence can be placed in the findings of systematic reviews of qualitative studies (PDF) [file pmed.1001187.s009.pdf]

Table S4: Assessing how much confidence can be placed in the findings of systematic reviews of qualitative studies[a]

**Key questions**
1. Did the review address an appropriate policy or management question?
2. Was a clear and appropriate explanation provided for the search approach used?
3. Were the criteria used to select studies appropriate?
4. Was the approach used to appraise the reliability of the included studies appropriate?
5. Was an appropriate approach used to analyse the findings of the included studies?
6. Does the review provide a coherent explanation of the issue of phenomenon under consideration?

Source: adapted from [1]

[a] A large number of tools are available to assess the quality of primary qualitative research (for example, [2,3]), and the principles underlying these tools may also be useful when assessing systematic reviews of qualitative studies.

References

1. Lewin S, Oxman AD, Lavis JN, Fretheim A (2009) SUPPORT Tools for evidence-informed health Policymaking (STP) 8: Deciding how much confidence to place in a systematic review. Health Res Policy Syst 7 Suppl 1: S8.

2. Spencer, L., Ritchie, J., Lewis, J., and Dillon, L. (2003) Quality in Qualitative Evaluation: A framework for assessing research evidence. England: Government Chief Social Researcher's Office.

3. Critical Appraisal Skills Programme (2006) 10 questions to help you make sense of qualitative research. England: Public Health Resource Unit.
