# Supplementary material for: Guidance for Evidence-Informed Policies about Health Systems: Assessing How Much Confidence to Place in the Research Evidence
Source: PLoS Med. 2012 Mar 20;9(3):e1001187. doi: 10.1371/journal.pmed.1001187 (PMC3308931; doi:10.1371/journal.pmed.1001187)
Supplement: Table S5 — Definitions of the quality of evidence categories within GRADE (PDF) [file pmed.1001187.s010.pdf]

Table S5: Definitions of the quality of evidence categories within GRADE

**High**	We are very confident that the true effect lies close to that of the estimate of the effect	⊕⊕⊕⊕
**Moderate**	We are moderately confident in the effect estimate: The true effect is likely to be close to the estimate of the effect, but there is a possibility that it is substantially different	⊕⊕⊕○
**Low**	Our confidence in the effect estimate is limited: The true effect may be substantially different from the estimate of the effect	⊕⊕○○
**Very low**	We have very little confidence in the effect estimate: The true effect is likely to be substantially different from the estimate of effect	⊕○○○

Source: [1]

References

1. Balshem H, Helfand M, Schunemann HJ, Oxman AD, Kunz R, Brozek J, Vist GE, Falck-Ytter Y, Meerpohl J, Norris S, Guyatt GH (2011) GRADE guidelines: 3. Rating the quality of evidence. J Clin Epidemiol 64: 401-406.
