# Supplementary material for: Guidance for Evidence-Informed Policies about Health Systems: Assessing How Much Confidence to Place in the Research Evidence
Source: PLoS Med. 2012 Mar 20;9(3):e1001187. doi: 10.1371/journal.pmed.1001187 (PMC3308931; doi:10.1371/journal.pmed.1001187)
Supplement: Table S6 — Example of factors affecting decisions about strength of recommendations—Changes in user fees in low- and middle-income countries (PDF) [file pmed.1001187.s011.pdf]

Table S6: Example of factors affecting decisions about strength of recommendations – Changes in user fees in low and middle income countries

<table>
<tr><td colspan="3">Population: Service users and health systems in low and middle income countries (LMICs)
Intervention: Changes in user fees, e.g. introducing, varying or removing user fees
Comparison: No user fees / no removal of user fees
Outcome: Use of health services</td></tr>
<tr><td>Key factors – is there uncertainty regarding:</td><td>Decision regarding whether there is uncertainty (yes / no)</td><td>Explanation of the decision made</td></tr>
<tr><td>Quality of evidence</td><td>Yes</td><td>All of the evidence was of very low quality (GRADE) and the studies showed mixed results on the use of health services</td></tr>
<tr><td>Balance of benefits versus harms and burdens</td><td>No</td><td>Review shows that people’s use of preventive healthcare services decreased and use of curative services generally decreased, which may increase inequities in access to services. However, when quality improvements were made to the health services at the same time as fees were introduced, people’s use of curative services increased</td></tr>
<tr><td>Acceptability</td><td>Yes</td><td>User fees are generally not favoured by service users, in part because they increase out-of-pocket expenses, but may be seen as an important source of revenue by decision makers and local health care providers. However, providers may have reservations about the day-to-day implementation of user fees [1]</td></tr>
<tr><td>Resource use</td><td>No</td><td>Introducing user fees imposes direct costs on service users and administrative costs on health facilities. The latter may outweigh the revenues accrued</td></tr>
<tr><td>Feasibility (or local factors that influence the translation of evidence into practice)</td><td>Yes</td><td>The administration of user fees may be a significant additional burden in under-resourced primary health care services and may also create tensions in provider-user relations [1]</td></tr>
<tr><td colspan="3">Recommended options for consideration
This assessment of evidence within a wider health system context might result in the following recommended options for consideration:
• Option 1: Where user fees are part of existing financing arrangements; there is strong political commitment to achieving universal access to care; and user fees are not favoured by users or health care providers:
    o Strong recommendation to remove user fees (i.e. there is confidence that the desirable effects of user fees do not outweigh the undesirable effects).
• Option 2: Where user fees are part of existing financing arrangements; there is little political commitment to achieving universal access to care; alternative financing arrangements may be difficult to implement; and there is strong opposition among providers to their removal and a reasonable likelihood that formal user fees may be replaced by informal user fees:
    o Conditional recommendation to remove user fees, linked to developing alternative financing arrangements (i.e. the desirable effects of removing user fees probably outweigh the undesirable effects but there is uncertainty).</td></tr>
</table>

Source: This table draws on evidence from [2]

References:

1. Ridde V, Morestin F (2011) A scoping review of the literature on the abolition of user fees in health care services in Africa. Health Policy Plan 26: 1-11.

2. Lagarde M, Palmer N (2011) The impact of user fees on access to health services in low- and middle-income countries. Cochrane Database Syst Rev CD009094.
