# Supplementary material for: Guidance for Evidence-Informed Policies about Health Systems: Assessing How Much Confidence to Place in the Research Evidence
Source: PLoS Med. 2012 Mar 20;9(3):e1001187. doi: 10.1371/journal.pmed.1001187 (PMC3308931; doi:10.1371/journal.pmed.1001187)
Supplement: Table S7 — Example of factors affecting decisions about strength of recommendations—Continuing education programmes for rural health workers to support their retention (PDF) [file pmed.1001187.s012.pdf]

Table S7: Example of factors affecting decisions about strength of recommendations – Continuing education programmes for rural health workers to support their retention

**Population:** Health workers in rural or remote areas<br>**Intervention:** Continuing education and professional development programmes<br>**Comparison:** No programmes<br>**Outcome:** Retention of health workers in rural areas		
**Key factors – is there uncertainty regarding:**	**Decision regarding whether there is uncertainty (yes / no)**	**Explanation of the decision made**
Quality of evidence	Yes	The evidence was of low quality (GRADE). There is indirect evidence that continuing education programmes influence the desire to remain in rural practice
Balance of benefits versus harms and burdens	No	• Benefits: In addition to facilitating knowledge acquisition and sharing, continuing education may also improve professional networking and reduce isolation.<br>• Disadvantages: programmes may be difficult to establish because of infrastructure and equipment requirements and lack of training capacity
Values and preferences	No	Such programmes likely to be valued by rural providers, particularly if linked to improved supportive supervision and career paths
Resource use	Yes	• Investments are needed to establish these programmes, e.g. to ensure internet access for online distance learning<br>• Travel costs and the costs of temporary replacements for staff attending training may need to be budgeted for<br>• Requires tutors and supervisors for both on-site and distance learning approaches
Feasibility (or local factors that influence the translation of evidence into practice)	Yes	• Distance learning may require internet access and availability of equipment<br>• Continuing education needs to be linked to career paths to be more attractive to health workers
**Recommended options for consideration**<br>*This assessment of evidence within a wider health system context might result in the following recommended options for consideration:*<br>• Option 1: Where the capacity to deliver appropriate training is available; resources are available to establish and maintain the programme; and there is strong support from rural providers and managers:<br>o Conditional recommendation to introduce continuing education programmes, in the context of evaluation (i.e. the desirable effects of introducing such programmes probably outweigh the undesirable effects but there is uncertainty and evaluation is needed to improve the evidence base for future decision making).<br>• Option 2: Where the capacity to deliver appropriate training in rural areas is very limited; resources to establish and maintain the programme are unlikely to be sustained; and there are concerns that the failure to sustain such programmes will further demoralize rural health workers:<br>o Conditional recommendation not to introduce continuing education programmes (i.e. the undesirable effects of introducing such programmes probably outweigh the desirable effects but there is uncertainty).		

Source: This table draws on evidence from [1]

References

1. WHO (2010) Increasing access to health workers in remote and rural areas through improved retention: global policy recommendations. Geneva: World Health Organization.
